# Testosterone Coordinates Gene Expression Across Different Tissues to Produce Carotenoid-Based Red Ornamentation

**DOI:** 10.1093/molbev/msad056

**Published:** 2023-03-13

**Authors:** Sarah Khalil, Erik D Enbody, Carolina Frankl-Vilches, Joseph F Welklin, Rebecca E Koch, Matthew B Toomey, Simon Yung Wa Sin, Scott V Edwards, Manfred Gahr, Hubert Schwabl, Michael S Webster, Jordan Karubian

**Affiliations:** Department of Ecology and Evolutionary Biology, Tulane University, New Orleans, LA, USA; Department of Ecology and Evolutionary Biology, Cornell University, Ithaca, NY, USA; Fuller Evolutionary Biology Program, Lab of Ornithology, Ithaca, NY, USA; Department of Biological Engineering, University of California Santa Cruz, Santa Cruz, CA, USA; Department of Behavioural Neurobiology, Max Planck Institute for Biological Intelligence, Seewiesen, Germany; Department of Biology, University of Nevada, Reno, Reno, NV, USA; Department of Biological Sciences, The University of Tulsa, Tulsa, OK, USA; Department of Biological Sciences, The University of Tulsa, Tulsa, OK, USA; School of Biological Sciences, The University of Hong Kong, Hong Kong, China; Department of Organismic and Evolutionary Biology, Harvard University, Cambridge, MA, USA; Museum of Comparative Zoology, Harvard University, Cambridge, MA, USA; Department of Organismic and Evolutionary Biology, Harvard University, Cambridge, MA, USA; Museum of Comparative Zoology, Harvard University, Cambridge, MA, USA; Department of Behavioural Neurobiology, Max Planck Institute for Biological Intelligence, Seewiesen, Germany; School of Biological Sciences, Washington State University, Pullman, WA, USA; Macaulay Library, Cornell Laboratory of Ornithology, Ithaca, NY, USA; Department of Neurobiology and Behavior, Cornell University, Ithaca, NY, USA; Department of Ecology and Evolutionary Biology, Tulane University, New Orleans, LA, USA

**Keywords:** pigmentation, androgens, gene expression, ornamental coloration, ketocarotenoids

## Abstract

Carotenoid pigments underlie most of the red, orange, and yellow visual signals used in mate choice in vertebrates. However, many of the underlying processes surrounding the production of carotenoid-based traits remain unclear due to the complex nature of carotenoid uptake, metabolism, and deposition across tissues. Here, we leverage the ability to experimentally induce the production of a carotenoid-based red plumage patch in the red-backed fairywren (*Malurus melanocephalus*), a songbird in which red plumage is an important male sexual signal. We experimentally elevated testosterone in unornamented males lacking red plumage to induce the production of ornamentation and compared gene expression in both the liver and feather follicles between unornamented control males, testosterone-implanted males, and naturally ornamented males. We show that testosterone upregulates the expression of *CYP2J19*, a gene known to be involved in ketocarotenoid metabolism, and a putative carotenoid processing gene (*ELOVL6*) in the liver, and also regulates the expression of putative carotenoid transporter genes in red feather follicles on the back, including *ABCG1.* In black feathers, carotenoid-related genes are downregulated and melanin genes upregulated, but we find that carotenoids are still present in the feathers. This may be due to the activity of the carotenoid-cleaving enzyme BCO2 in black feathers. Our study provides a first working model of a pathway for carotenoid-based trait production in free-living birds, implicates testosterone as a key regulator of carotenoid-associated gene expression, and suggests hormones may coordinate the many processes that underlie the production of these traits across multiple tissues.

## Introduction

Carotenoid pigments, which are responsible for many of the vivid red, orange, and yellow colors observed in vertebrates, are core components involved in the evolution of social signals. Among vertebrates, carotenoid-based colors are widely believed to serve as honest indicators of quality, motivating classic research surrounding the adaptive benefits of sexual signaling (e.g., Hill and McGraw 2006; Svensson and Wong 2011) and inspiring some of the most well-known hypotheses for sexual selection (e.g., handicap principal [Zahavi 1975], good genes hypothesis [von Schantz et al. 1999; Reinhold 2004]). Yet many aspects of the underlying mechanisms of carotenoid color production, including its genetic basis, remain relatively uncharacterized (Toews et al. 2017) (though see Badyaev et al. (2015), Balenger et al. (2015), and Morrison and Badyaev (2018)). Identifying the genetic basis of carotenoid coloration has been particularly challenging because of the complex interplay of tissue types and processes thought to underlie the expression of this type of coloration: in birds, carotenoids must first be obtained from their diet, transported through the body, potentially metabolized into alternative pigment forms (often in the liver), and potentially transported again before deposition in the final tissues (integument and feathers). This pathway requires complex coordination among different genes and cellular processes across multiple tissues.

Recent work identifying and describing the roles of a handful of genes important for carotenoid transport (Toomey et al. 2017), carotenoid metabolism (Lopes et al. 2016; Mundy et al. 2016; Toomey, Marques, et al. 2022), and carotenoid cleavage (Eriksson et al. 2008; Gazda, Toomey, et al. 2020; Enbody et al. 2021) has provided insights into the biochemical mechanisms underlying this important signaling modality. However, most of this work has been done in domesticated lab animals and/or in a single tissue type, and much remains to be discovered about the multi-tissue genetic pathways likely to underlie this signal production. Moreover, although there is good evidence that hormones such as testosterone mediate phenotypic variation through changes in gene expression (Ketterson and Nolan 1992; Cox et al. 2016; Enbody et al. 2022), there have been few studies that directly link endocrine and genetic mechanisms of carotenoid production (Khalil et al. 2020).

**Fig. 1.**
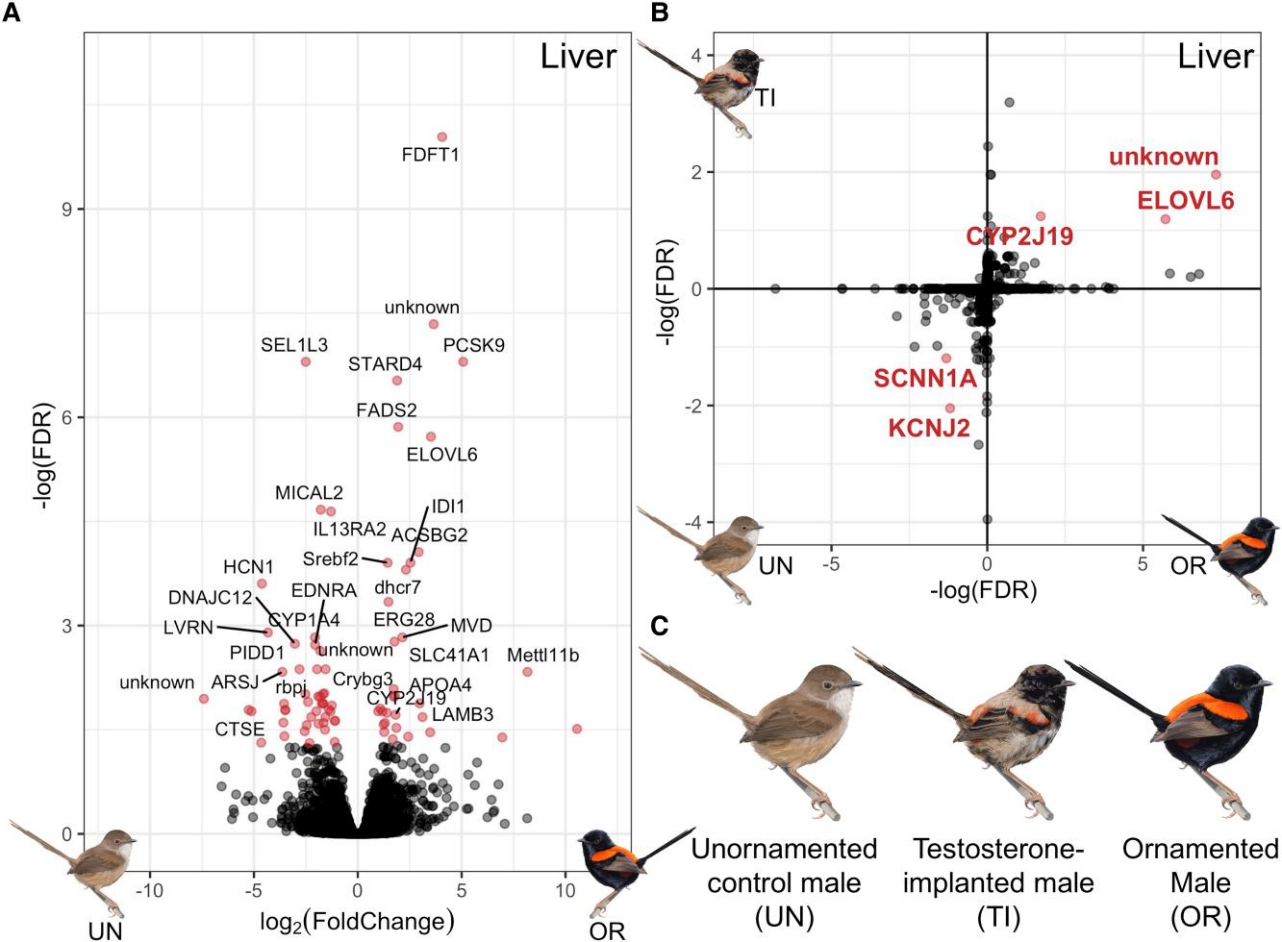
(*A*) Volcano plot of differential expression of genes in the liver between unornamented males and ornamented males, with significantly overexpressed genes (adjusted *P* < 0.05) plotted in red. Not all genes are labeled due to space. (*B*) Differential expression of shared genes in the liver between control unornamented (UN) and ornamented (OR) males (*x*-axis) and between unornamented and testosterone-implanted (TI) males (*y*-axis); genes that are significantly overexpressed (adjusted *P* < 0.1) in both ornamented males and testosterone-implanted males are located in the top right quadrant, and genes that are significantly overexpressed in unornamented males are in the bottom left quadrant. Significantly differentially expressed genes are colored and labeled in red. The unit on each axis is –log10(FDR) (FDR), an adjusted measure of significance that takes account for direction of expression. (*C*) Depiction of the three phenotypes used in the differential expression analysis. Unornamented males have a brown back and cream chest, testosterone-implanted males were just starting to molt in red back feathers and black chest feathers at the time of collection, and ornamented males have a red back and black chest.

The red-backed fairywren (*Malurus melanocephalus*) provides a useful study system in which to identify the endocrine and genetic control of an important carotenoid-based signal. In this Australian songbird, males can flexibly express either ornamental black body plumage with a carotenoid-based red dorsal feather patch or female-like unornamented brown plumage with a cream-colored chest and no dorsal red patch (Rowley and Russel 1997; Rowe and McGraw 2008; fig. 1*C*). Expression of unornamented versus ornamented plumage depends on several factors including age and breeding status (Webster et al. 2008; Khalil et al. 2020; Welklin et al. 2021), and both correlative and experimental studies have shown that production of the eponymous red patch in males is mediated by increased levels of circulating testosterone (Lindsay et al. 2009, 2011, 2016). Circulating carotenoid levels also are associated with plumage phenotype, in that ornamented males have much higher levels of metabolized ketocarotenoids circulating in their blood than do unornamented males (Khalil et al. 2020).

Experimental and correlative evidence indicates strong female preference for males with redder patches (Webster et al. 2008; Baldassarre and Webster 2013), demonstrating that ornamented plumage is a sexually selected trait. Sexual selection drives the introgression of redder plumage between subspecies that vary between orange and red plumage (Baldassarre et al. 2014). Thus, carotenoid-based variation mediates both short-term (intra-population) and long-term evolutionary change (interpopulation). Here, we used testosterone implants to experimentally induce production of the red plumage patch in free-living birds. This in turn allowed us to identify differences in gene expression associated with carotenoid metabolism, transport, and feather pigmentation between naturally unornamented males, testosterone-implanted treatment males, and naturally ornamented males and to test the hypothesis that testosterone coordinates the expression of those genes and the regulation of pathways across tissues.

## Results and Discussion

### Carotenoid Pigment Metabolism in the Liver

To assess differential gene expression between different plumage phenotypes, we captured free-living red-backed fairywrens on our long-term study site in Samsonvale, Australia (Webster et al. 2010) in mist nets, and generated RNA-seq libraries from collected tissues and plucked molting feathers that were stored them in RNAlater until RNA extraction (following Enbody et al. 2022). First, we compared gene expression in the liver of naturally ornamented males to naturally unornamented males (*n* = 3 for each phenotype) due to previous work suggesting the importance of the liver in carotenoid metabolism (Lopes et al. 2016; Mundy et al. 2016; Khalil et al. 2020) and found 76 differentially expressed genes. The most upregulated genes (with the largest –log[FDR] values) in ornamented males included *FDFT1*, *PCSK9*, *STARD4*, *FADS2*, *ELOVL6*, and *ACSBG2*, which are known to be involved in lipid transport and processing (Seidah et al. 2012; Bateman et al. 2021; fig. 1*A*). Carotenoids are lipophilic, and other genes involved in carotenoid processing in birds were initially described in humans as having lipid-associated functions (e.g., *SCARB1*; Toomey et al. 2017), so these genes may also contribute to carotenoid processing in the liver that leads to the production of red feathers. In addition, *CYP2J19*, one of the few genes with a known carotenoid-metabolizing function in birds (Lopes et al. 2016; Mundy et al. 2016) was also upregulated in the liver of ornamented males. Specifically, *CYP2J19* encodes a key enzyme in the carotenoid ketolase pathway that metabolizes yellow dietary carotenoids into the red ketocarotenoid pigments that underlie most red feather coloration in birds, including in red-backed fairywrens (Rowe and McGraw 2008; Khalil et al. 2020; Toomey, Marques, et al. 2022). However, CYP2J19 does not act alone, and a recent study has identified a second enzyme, BDH1L, that is necessary for ketocarotenoid metabolism (Toomey, Marques, et al. 2022). Consistent with this two-step mechanism, we found that *BDH1L* is expressed in the liver of red-backed fairywrens, but not differentially expressed between any of the phenotype comparisons. Since *CYP2J19* is differentially expressed between plumage phenotypes in red-backed fairywrens, but *BDH1L* is not, this suggests that *CYP2J19* expression levels determine whether ketocarotenoid metabolism occurs, though both genes are essential for this metabolism to happen.

To understand the role of testosterone in mediating these gene expression differences that produce red ornamentation, we experimentally elevated testosterone in free-living unornamented males (*n* = 3) via subcutaneous testosterone implants, plucked a small number of feathers to induce new growth, and then released the birds. We recaptured these same individuals 10–12 days after implantation for tissue collection. At the time of collection, testosterone-implanted males had begun to molt new red feathers on their backs and black feathers on the rest of the body. We compared the subset of genes in the liver that were naturally differentially expressed between unornamented and ornamented males to those induced through experimental testosterone treatment (fig. 1*B*). We found 15 genes differentially expressed between testosterone-implanted and control unornamented males, five of which were genes also differentially expressed between naturally unornamented and ornamented males (three up and two down, 33% of all testosterone-induced differentially expressed genes, adjusted *P* value < 0.1 for both comparisons). Two of these shared upregulated genes were *CYP2J19* and *ELOVL6*. These findings suggest that testosterone induces changes in expression of a small number of genes in the liver (15 out of 16,963 total genes, 0.09% of all fairywren genes examined) that likely have major effects on carotenoid production.

### Carotenoid Pigment Transport into Feathers

**Fig. 2.**
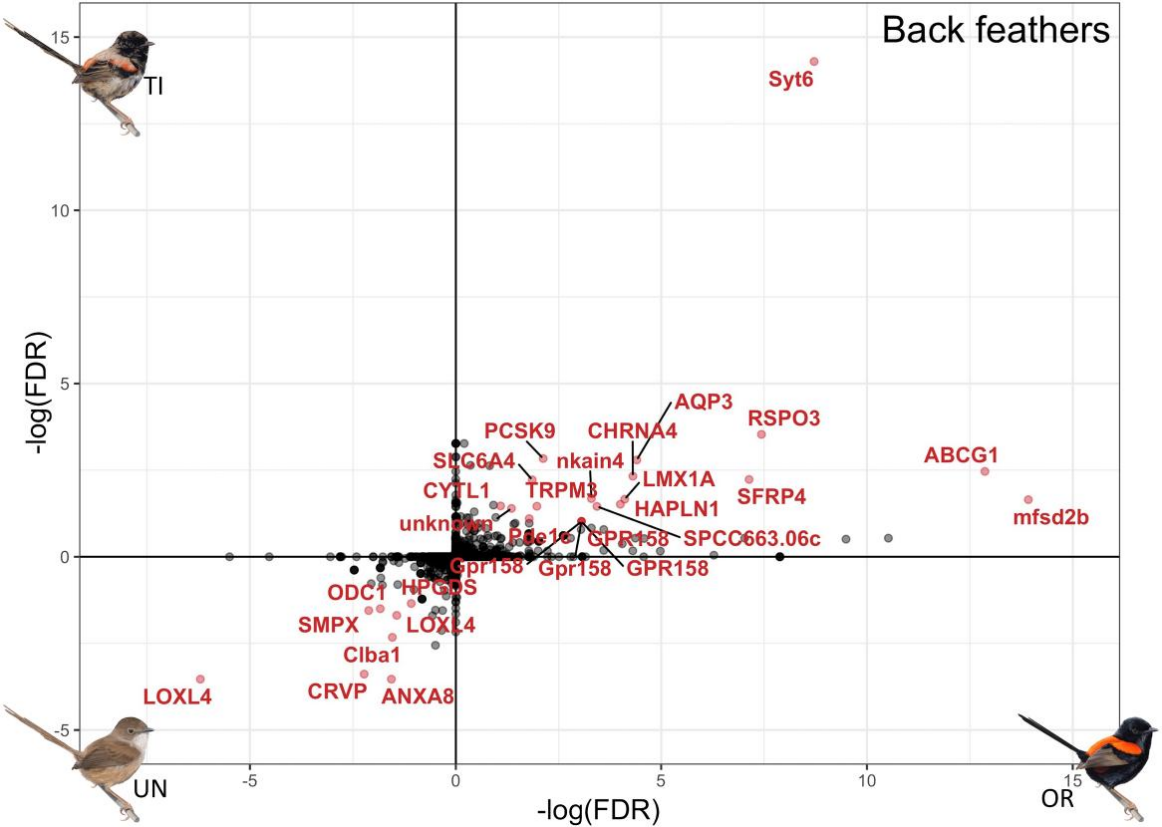
Differential expression of shared genes in back feathers between control unornamented (UN) and ornamented (OR) males (*x*-axis) and between unornamented and testosterone-implanted (TI) males (*y*-axis); genes that are significantly overexpressed (adjusted *P* < 0.1) in red feathers (in both ornamented males and testosterone-implanted males) are located in the top right quadrant, and genes that are significantly overexpressed in brown feathers (unornamented males) are in the bottom left quadrant. Significantly differentially expressed genes are colored and labeled in red.

Metabolized carotenoids in the liver must be transported to feathers to produce the red phenotype, which may involve other gene networks. We compared gene expression in the growing back feathers between naturally ornamented males (red feathers) to unornamented males (brown feathers) and found 107 differentially expressed genes between these two phenotypes (supplementary fig. S4*A*, Supplementary Material online). Among the 35 genes upregulated in ornamented males was *SCARB1*, a gene that helps promote cellular uptake of carotenoids in birds, including in the skin where feathers develop (Toomey et al. 2017). To evaluate if testosterone induces this component of ornamentation, we again compared the genes naturally differentially expressed between naturally unornamented and ornamented feathers to those induced through the experimental testosterone treatment. We found overlap in 22 out of the 52 genes differentially expressed between testosterone-implanted and control unornamented males (7 down and 15 up, 42% of all testosterone-induced differentially expressed genes, adjusted *P* value < 0.1 for both comparisons; fig. 2). Of the 15 genes upregulated in both ornamented and testosterone-implanted males, *RSPO3* and *SFRP4* are involved in the Wnt-signaling pathway, a pathway commonly implicated in feather production and coloration in birds (Chang et al. 2004; Toews et al. 2016). Five other genes (33% of testosterone-induced upregulated genes) were associated with transmembrane and vesicle-mediated transport functions (including *SYT6*, *AQP3*, and *TRPM3*), and two of those (*ABCG1* and *MFSD2B*) were specifically associated with lipid transport (Vaughan and Oram 2005; Vu et al. 2017). Another gene in the ATP-binding cassette (ABC) gene family, *ABCA1*, has previously been described in Wisconsin hypoalpha mutant (WHAM) chickens as having functions related to high-density lipoprotein transport; a mutation in this gene leads to a reduced level of carotenoids in blood and tissues (Attie et al. 2002; Connor et al. 2007). In mammals, the ABCG gene family is associated with cholesterol efflux (Sun et al. 2021). Therefore, *ABCG1* may serve a qualitatively similar function in red-backed fairywrens, the transport of carotenoids into red feathers. It is also important to note that *CYP2J19* was not differentially expressed in any brown versus red feather comparison, suggesting that dietary carotenoids are exclusively metabolized into ketocarotenoids in the liver of red-backed fairywrens and then transported into feathers, potentially via transport processes regulated by the above-identified transport genes.

### Melanin Production and Carotenoid Breakdown in Feathers

**Fig. 3.**
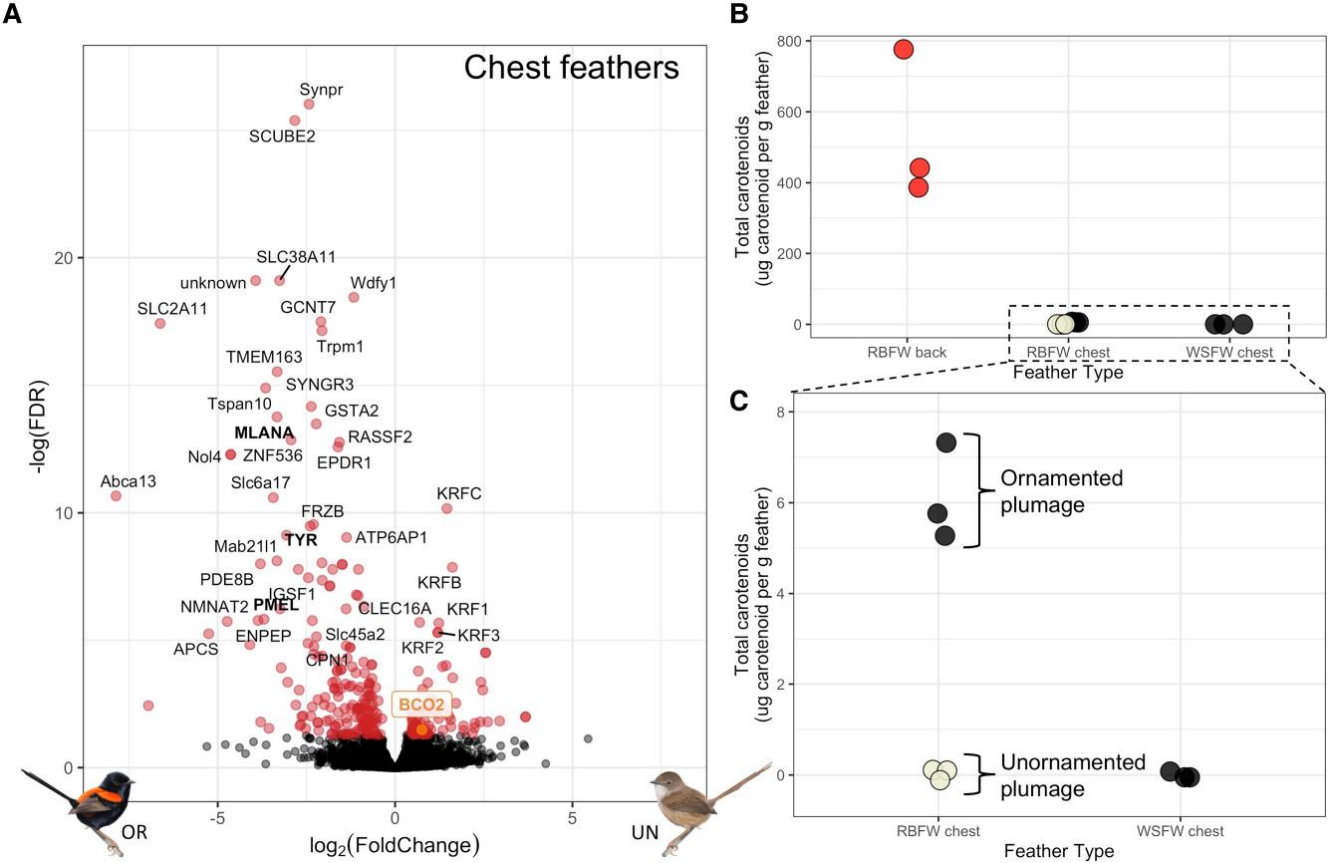
(*A*) Volcano plot of differential expression of genes in chest feathers between ornamented (OR) males (black feathers) and control unornamented (UN) males (cream feathers), with significantly overexpressed genes (adjusted *P* < 0.05) plotted in red. Genes labeled in bold black are part of the melanogenesis pathway, and BCO2 is highlighted in orange for ease of viewing. (*B*) Total carotenoid concentration in red-backed fairywren (*Malurus melanocephalus*) back feathers, red-backed fairywren chest feathers, and white-shouldered fairywren (*M. alboscapulatus*) chest feathers. The color of the points represents the color of those feathers (red, cream, and black). Note RBFW chest feathers were sampled from both ornamented (black) and unornamented (cream) males. (*C*) A zoom-in of figure 4*B* to see differences in black and cream RBFW chest feathers. There were no carotenoids detected in unornamented (cream) RBFW chest or WSFW chest feathers and were therefore coded as “0” for these plots.

We sampled and compared gene expression of the black chest feathers of naturally ornamented males, which naturally have higher testosterone levels, to the cream chest feathers of unornamented males to test whether testosterone similarly regulated other pigment-related genes, and found 258 differentially expressed genes between these two phenotypes (fig. 3*A*). Upregulated genes in ornamented males included several known melanogenesis genes, including *MLANA*, *TYR*, and *PMEL* (Fukai et al. 1995; Berson et al. 2001; Hoashi et al. 2005), which was expected as we were comparing cream feathers to melanized black feathers. We also found that the gene *BCO2*, a carotenoid-cleaving enzyme, was upregulated in cream chest feathers of unornamented males, indicative of carotenoids being actively broken down during their chest feather production, as BCO2 is a carotenoid-cleaving enzyme responsible for the degradation of carotenoids in birds and other animals (Våge and Boman 2010; Lehnert et al. 2019; Gazda, Toomey, et al. 2020; Price-Waldman and Stoddard 2021). Reduced expression of this gene in the black chest feathers of ornamented males suggests the presence of carotenoids in black feather follicles. To test this, we performed high-performance liquid chromatography (HPLC) to quantify carotenoids of red ornamented red-backed fairywren (*M. melanocephalus*) back feathers, black ornamented *M. melanocephalus* chest feathers, and cream unornamented *M. melanocephalus* chest feathers. We also selected black ornamented white-shouldered fairywren (*Malurus alboscapulatus*) chest feathers (*n* = 3 per feather type) as a closely related outgroup that we predicted would not accumulate carotenoids (as they have no red feathers). As expected, we found high levels of carotenoids in the red *M. melanocephalus* back feathers (mean = 535 µg of carotenoids per gram of feather) and were not able to detect any carotenoids in either the cream *M. melanocephalus* or the black *M. alboscapulatus* chest feathers (fig. 3*B*and*C*). However, we did find small but detectable amounts of carotenoids in the black *M. melanocephalus* chest feathers (mean = 6 µg of carotenoids per gram of feather). This suggests that the black feathers found across most of the body of ornamented male red-backed fairywrens (or at least the ones on the chest) may contain carotenoids hidden behind the black melanin pigments, as has recently been reported in other birds (McCoy et al. 2021), and that carotenoids are actively broken down during the production of cream chest feathers.

### Phenotypic Associations with Gene Expression Networks in Feather Follicles

**Fig. 4.**
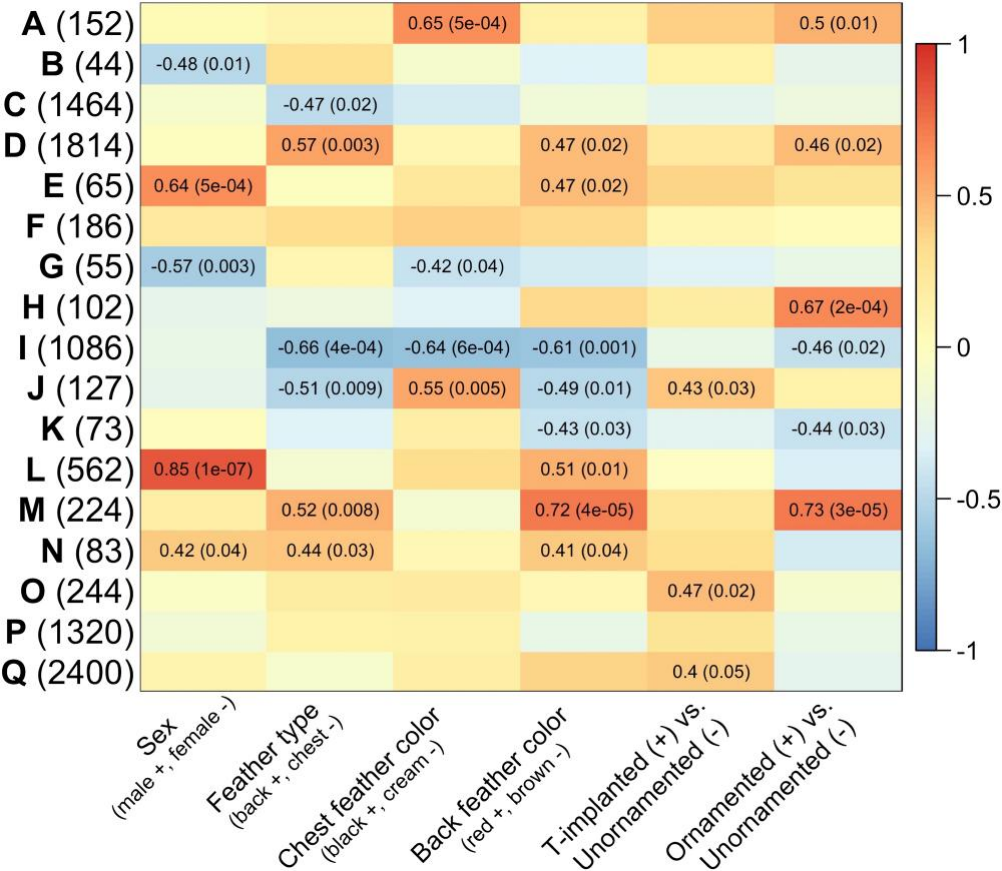
Module-trait correlations from WGCNAs for feathers. Modules are indicated by letters, and the number of genes per module is in parentheses. For each module eigengene, heatmaps present correlation coefficients and associated *P* values in parentheses for *P* ≤ 0.05. Parentheses on the *x*-axis represent how the bimodal traits are coded in terms of the coefficient values. Module “J” was enriched for several pigmentation functions, specifically associated with melanin production. Module “K” was enriched for lipid metabolic functions. Module “M” was enriched for cellular component disassembly functions and protein modification functions, which are associated with actin filament formation important for feather formation. More details of functions associated with these and other modules can be found in text.

We used weighted gene coexpression network analysis (WGCNA) to identify modules of coregulated testosterone-sensitive genes (Langfelder and Horvath 2008). We used phenotype as our contrast (e.g., female vs. control unornamented male vs. T-implanted male vs. ornamented male), and combined both back and chest feathers in our analysis. WGCNA identified 17 modules, most of which were differentially regulated in relation to at least one trait (fig. 4). To understand how these modules may be functionally related to these traits, we used gene ontology (“GO”) analysis to identify shared functional properties of genes involved in each module. We used WebGestalt (Liao et al. 2019) to conduct GO enrichment analysis and compared enrichment results using functional databases for both *Homo sapiens* (human) and *Gallus gallus* (chicken). Results were similar using each reference database, but the human reference database was larger and often included more specific functional categories. We identified genes in each module with the highest network connectivity assessed by module membership and only included genes in GO enrichment analysis that had a module membership ≥0.6 (supplementary tables S8 and S9, Supplementary Material online).

In this analysis, Module “K” was correlated with back feather color and was enriched for lipid metabolic functions in both human and chicken databases, as well as more specific categories of regulation of lipid metabolic processes and isoprenoid metabolic processes in the human database. As mentioned previously, carotenoids have similar structure to lipids, and therefore genes with associated lipid functions may also serve to provide carotenoid processing functions in feathers. Similarly, Module “M” was also correlated with back feather color but did not have any significantly enriched functions. However, some genes with high module membership included *SCARB1*, a known lipid transporter important for carotenoid-based coloration (Toomey et al. 2017), as well as a related gene *SCARB2*, and another lipid transport gene *ABCA4*. These results, in combination with the differential expression results from back feathers, further emphasize the potential importance of continuing to explore and characterize lipid processing genes for carotenoid coloration.

Module “I” was correlated with several traits including chest feather color, with genes being upregulated in cream chest feathers. Though there were no significantly enriched GO terms in this module using the human database, the module was enriched for cellular component disassembly and protein modification by small protein conjugation or removal in the chicken database. Some genes associated with these GO terms are important for actin filament formation (Bateman et al. 2021), which may be important for feather bud formation (Kim et al. 2005) and phenotype differences in feathers (Leskinen et al. 2012). Other genes with high module membership included several avian keratin genes (*KRF1*, *KRF2*, *KRF3*, *KRFA*, *KRFB*, *KRFC*, and *KRFD*), which are known to be important for feather production, especially feathers that contain little or no pigmentation (e.g., white and cream colors, Ng et al. 2014). Module “J” was also correlated with both back and chest feather color, as well as testosterone treatment (T-implanted vs. unornamented individuals, fig. 4). This module was enriched for a broad pigmentation category in chicken and multiple, more specific, pigmentation-linked functions involving melanin biosynthetic processes in the human GO database. Genes with high module membership included *PMEL*, *MLANA*, *TYRP1*, *OCA2*, *SLC24A5*, *MREG*, *RAB27A*, and *RAB32*, which are genes known to be involved in melanin production underlying black and brown coloration (Damek-Poprawa et al. 2009; Bellono et al. 2014; Yu et al. 2018; Zheng et al. 2020; McNamara et al. 2021; Yu et al. 2021). Genes and pathways involved in melanogenesis are expected to be associated with feather color differentiation in this system, but it is also interesting to see this module correlated with testosterone treatment, as there has been mixed results around the importance of testosterone for melanin-based feather production in red-backed fairywrens (Lindsay et al. 2016).

Several modules were correlated with sex (fig. 4), and although there were significantly enriched GO terms in some of these modules (supplementary tables S9 and S10, Supplementary Material online), it is unclear how these functions may be related to sex differences. Because sex differences were not the focus of this study, we did not further explore the genes or functions in these modules. However, understanding the genetic basis of sex differences and the female-like plumage phenotype of unornamented males does warrant further investigation (Gazda, Araújo, et al. 2020) but would require a more focused sampling design.

## Conclusion

Here, we used an experiment on free-living male red-backed fairywrens to provide new insights into the interplay of specific genes and the associated endocrine control underlying the production of a carotenoid-based plumage signal. We find that the liver is a central site for carotenoid metabolism, corroborating previous tissue-specific work in captive populations of other birds (Del Val et al. 2009; Ge et al. 2015; Lopes et al. 2016; Mundy et al. 2016). We also show that testosterone upregulates the expression of a key gene in ketocarotenoid production (*CYP2J19*) and a putative gene involved in carotenoid processing (*ELOVL6*) in the liver of red-backed fairywrens. Testosterone also regulates the expression of putative carotenoid transport genes in follicles of red feathers, including *ABCG1*, a gene in the same gene family as one associated with carotenoid transport in chickens (Connor et al. 2007). Finally, we show that *BCO2*, a gene associated with carotenoid breakdown, is upregulated in unornamented cream chest feathers compared with ornamented black chest feathers. Low *BCO2* expression in black feathers is likely responsible for the presence of carotenoids in black feathers, which may be caused by a conflict with this carotenoid-cleaving enzyme and the production of the carotenoid-based red back feathers.

**Fig. 5.**
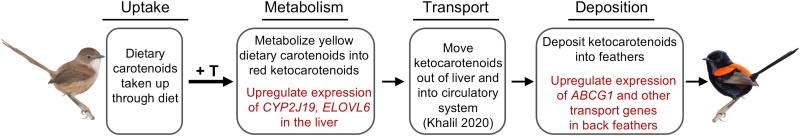
A working mechanistic model for how testosterone regulates a pathway that leads to the acquisition of carotenoid-based plumage ornamentation in male red-backed fairywrens. Processes that have been identified in this work are highlighted in red.

Identifying the genetic basis of carotenoid coloration has been challenging in part because of the complexity underlying the processing, transport, and deposition of carotenoids on an organismal level. Here, we were able to leverage the testosterone dependence of carotenoid-based signal production in red-backed fairywrens to experimentally identify new putative genes of carotenoid-based pigmentation and further support the function of already described carotenoid genes. In doing so, we connect several of the complex processes that underlie carotenoid coloration, by showing how testosterone triggers a pathway in which carotenoids are first metabolized in the liver, transported presumably via blood circulatory systems (Khalil et al. 2020) to sites of feather production, and then deposited or potentially broken down in molting feathers (fig. 5). Though testosterone may not activate carotenoid-based coloration in all birds (Peters et al. 2004, 2012), this work provides a first working model describing some of the underlying genetic and endocrine mechanisms of production of a carotenoid-based signal in a wild system, one with a priori evidence of sexual selection on the trait. This work also expands on the role of testosterone as a phenotypic integrator mediating the expression of several other traits, including soft-part coloration, sperm production, and behavior in red-backed fairywrens (Karubian 2002, 2008; Karubian et al. 2011; Barron et al. 2015). We thereby present new avenues of investigation into the proximate basis of carotenoid-based color expression and ultimately into the evolution and adaptive significance of these traits.

## Materials and Methods

### Testosterone Implantation and Liver Sample Collection

We collected samples from free-living red-backed fairywrens captured in mist nets at our long-term study site in Samsonvale, QLD, Australia (27°27′S, 152°85′E). We collected liver samples from four types of breeding red-backed fairywrens (testosterone-implanted males, unornamented control males, ornamented males, and females) in November 2017, as described below. First, three breeding unornamented males were implanted with testosterone (testosterone-implanted males), and three breeding unornamented males were implanted with sham implants (unornamented control males). At the time of initial capture and implantation, around ten feathers were plucked from the center of the back to induce feather replacement at that location, as described in (Khalil et al. 2020). Implants were composed of beeswax (73% by weight; Sigma-Aldrich, St. Louis, MO, USA) and hardened frozen peanut oil (24% by weight; ACROS Organics, NJ, USA) that were mixed in a water bath at 67°C. Once the beeswax/peanut oil mixture was melted, crystalline testosterone (3% by weight; Sigma-Aldrich, St. Louis, MO, USA) was dissolved in 2.5 µl of 200 proof ethanol (Fisher Bioreagants), and the testosterone suspension was then added to the wax mixture and stirred. The implants were formed by feeding partially solidified wax through the tip of a syringe that was cut so the diameter was 2 mm, resulting in implants of 2 × 3.2 mm weighing between 19.8 and 20.7 mg. Testosterone concentration in the beeswax carrier was scaled to produce high physiological concentrations found in circulation during the breeding season (Lindsay et al. 2009).

Implants were inserted subcutaneously using forceps above the thigh into a small skin incision that was sealed with veterinary skin adhesive. After confirming that the incision was completely sealed and the bird was in good condition, the bird was released. Implanted birds (testosterone and sham) were recaptured 10–12 days postimplantation for sample collection, a time period that allowed for growth of pin feathers in the plucked plumage patches. Testosterone-implanted males molted in red pin feathers, whereas sham-implanted males molted in a mix of red and brown pin feathers, as many unornamented males do over the course of the breeding season (Lindsay et al. 2011, 2016). This is consistent with what had been observed in a previous feather-plucking study in this species (Karubian et al. 2011). We were unable to recapture one of the sham-implanted birds after implantation. Instead, we captured and obtained samples from one additional breeding unornamented male, who was not implanted, to include in our control group.

Following recapture, birds were immediately sacrificed by cervical dislocation. Body dissection was performed immediately, and the right lower lobe of the liver was removed, stored in 1 ml of RNAlater storage buffer (Thermo Fisher Scientific), and immediately placed on dry ice. Samples were stored at −80°C until RNA extraction. In addition to the three testosterone-implanted unornamented males and the three control unornamented males, we also collected liver samples from three ornamented breeding males and three breeding females (without implants). The sampling scheme is also described in supplementary figure S1, Supplementary Material online. We did not include auxiliary helpers in this experiment to avoid potentially confounding underlying differences in endocrine or genetic profiles that may exist between auxiliary nonbreeding versus breeding individuals (Lindsay et al. 2009). All sacrificed birds were seen paired with a male or female within the 2 weeks prior to sample collection, and all samples were collected within a period of 9 days. Circulating androgens were measured using an established radioimmunoassay protocol for this species (full methods in Lindsay et al. (2009) and Barron et al. (2015)); the intra-assay coefficient of variation was 8.68%. Testosterone-implanted birds were confirmed to have high concentrations of circulating androgens at the time of collection (mean = 3027 pg/ml, range = 2,198–4,065 pg/ml), which is within the natural range of androgens for breeding ornamented males in this species (Lindsay et al. 2009). Ornamented males also had similarly high levels of androgens at the time of collection (mean = 1,834 pg/ml, range = 1,124–2,925 pg/ml). We were unable to obtain samples to assay testosterone concentrations for control unornamented males or females. Nevertheless, previous measurements of androgens for unmanipulated unornamented males (mean concentration = 798 ± 174 pg/ml; Lindsay et al. 2011) and females (mean concentration = 191 ± 82 pg/ml; Lindsay et al. 2016) are considerably lower than our testosterone-implanted or ornamented male groups.

### Feather Sample Collection

We collected back and chest feather samples from nonbreeding red-backed fairywrens in June to July 2017, just prior to the breeding season. First, three 1-year-old unornamented males were implanted with testosterone, as described above. These males were recaptured 10–12 days after implantation for feather collection. From each individual, we used sterilized tweezers to collect two actively molting feathers from the center of the back just as the feather was erupting from the sheath and stored them together in 1 ml of RNAlater storage buffer (Thermo Fisher Scientific) and immediately placed them on ice.

We also collected feather samples from nonimplanted 1-year-old unornamented males, naturally ornamented males, and females. We sampled feathers opportunistically for these birds—if we caught a bird and found that they were molting feathers at the correct stage (just erupting from the sheath) and in the correct area (on the back), we collected and stored them in RNAlater. In total, we collected feather samples from three unornamented males implanted with testosterone, three unornamented males with no implantation, three ornamented males, and three females, similar to our sampling scheme for our liver collection. Though we collected molting feather samples from the same birds sampled for liver tissues in November 2017, we only had feather samples for implanted males and sham-implanted males, but none from females or ornamented males because they were not molting at that time. Instead, we chose to sequence feathers collected in June–July 2017 because they were all collected during the same nonbreeding season, and we would therefore limit confounding effects of comparing feather samples between nonbreeding and breeding seasons.

### Reference Genome Library Preparation, Sequencing, and Assembly

We requested a tissue sample of a male *M. melanocephalus* from Western Australia accessioned in the Museum of Comparative Zoology Ornithology collection (catalog number: 337443) and isolated genomic DNA using the DNeasy Blood and Tissue Kit (Qiagen, Hilden, Germany) following the manufacturer's protocol. We measured DNA concentration with a Qubit dsDNA HS Assay Kit (Invitrogen, Carlsbad, USA). We performed whole-genome library preparation, sequencing, and assembly (following Sin et al. 2020). In brief, a DNA fragment library of 220-bp insert size was prepared using the PrepX ILM 32i DNA Library Kit (Takara), and mate-pair libraries of 3-kb insert size were prepared using the Nextera Mate Pair Sample Preparation Kit (cat. No. FC-132-1001, Illumina). We performed DNA shearing for the fragment and mate-pair library preparations using Covaris S220. We used the 0.75% agarose cassette in the Pippin Prep (Sage Science) for size selection of the mate-pair library (target size 3 kb, “tight” mode). We then assessed fragment and mate-pair library qualities using the High Sensitivity D1000 ScreenTape for the Tapestation (Agilent) and High Sensitivity DNA Kit for the Bioanalyzer (Agilent), respectively, and quantified the libraries with qPCR (KAPA Library Quantification Kit) prior to sequencing. We sequenced the libraries on an Illumina HiSeq 2500 instrument (High Output 250 kit, PE 125-bp reads) at the Bauer Core facility at Harvard University to ∼71× coverage. We assessed the quality of the sequencing data using FastQC and removed adapters using Trimmomatic (Bolger et al. 2014). We assembled the genome using AllPaths-LG v52488 (Gnerre et al. 2011). We estimated the completeness of the assembled genome with BUSCO v2.0 (Simão et al. 2015) (supplementary table S1, Supplementary Material online).

### RNA-seq Library Preparation and Sequencing

To extract messenger RNA from tissue samples, we removed liver and feathers from the RNAlater buffer and homogenized either a ∼5-mm^3^ portion of liver or two feathers per sample, in a Qiagen TissueRuptor, and used a Qiagen RNAeasy minikit, following manufacturer's instructions. RNA quality was assessed with the Agilent 2100 Bioanalyzer Instrument (Model G2939A, Agilent Technologies RNA). Concentrations were measured with a Nanodrop 1000 spectrometer (Thermo Fisher Scientific). About 1 µg of total RNA per sample was used to construct RNA sequencing libraries using the TrueSeq RNA Sample Preparation Kit, v2 (Illumina Inc., San Diego, CA, USA). The resulting libraries were barcoded then sequenced on Illumina Hiseq 2500 and HiSeq 4000 systems. The sequencing protocol was set to high output mode with paired-end 50- or 75-bp reads, with an output of 60–100 million reads per sample.

### Preprocessing RNA Sequences

We first used Rcorrector (Song and Florea 2015) to correct for sequencing errors in Illumina RNA-seq reads and removed kmers with errors that were unfixable using a custom python script (https://github.com/harvardinformatics/TranscriptomeAssemblyTools/blob/master/FilterUncorrectabledPEfastq.py). We subsequently trimmed adaptors and removed low-quality reads (-q 5) using TrimGalore 0.4.4, which is a wrapper script around Cutadapt (Martin 2011). We next downloaded ribosomal RNA databases from Silva (Quast et al. 2013) for small subunit (Nr99) and large subunit (128) rRNA. We aligned reads to the concatenated rRNA database using BowTie2 2.3.3 (Langmead and Salzberg 2012), with the –very-sensitive-local option, and retained only those reads that did not map to the database.

### Transcript Alignment

We aligned transcripts to the red-backed fairywren reference genome using STAR (Dobin and Gingeras 2015) with annotations generated using MAKER. We ran STAR using the BjSJout function to remove spurious splice junctions, removed noncannonical reads, and using the default twopassMode. Lastly, we used STAR to count the number of reads per gene using the –quantMode GeneCounts function, which we used as input for differential testing.

### Differential Expression Analysis

We used the DESeq2 R package from Bioconductor (Love et al. 2014) to determine if the counts of genes differed between focal comparisons. DESeq2 uses negative binomial generalized linear models to determine if a given gene is expressed differently between treatments. We were interested in the transcriptional architecture of plumage color differences in feather and liver tissue, as well as how testosterone regulates the expression of these genes important for determining color differences. Therefore, we focused on a comparison identifying shared genes between natural transitions from unornamented to ornamented plumage and between unornamented plumage and testosterone implantation–induced ornamented plumage. Differentially expressed genes were tested for significance using a Wald test and adjusted using a Benjamini–Hochberg adjustment for multiple comparisons (as implemented by DESeq2; Love et al. 2014). We used the default parameter for false discovery rate (FDR) and adjusted *P* value cutoff of 0.05. When visualizing overlapping significant genes, we considered genes with an adjusted *P* value cutoff of 0.1 for both comparisons. Gene functions were identified by searching available literature and the UniProtKB database (Bateman et al. 2021).

### Weighted Gene Coexpression Networks and GO Enrichment Analyses

We used WGCNA to identify modules of coregulated testosterone-sensitive genes (Langfelder and Horvath 2008). We used phenotype as our contrast (e.g., female vs. control unornamented male vs. T-implanted male vs. ornamented male) and combined both back and chest feathers in our analysis. First, we filtered out genes with <10 normalized counts in 88% of samples, and then we used the 75% most variable genes, leaving 9981 genes. We built a signed hybrid network using a biweight midcorrelation (bicor) function and soft threshold power (*β*) = 8, in accordance with scale-free topology. We set minimum module size to 30, and modules with similar expression profiles were merged using Dynamic Tree Cut using a threshold of 0.25 since these genes are highly coexpressed. We then correlated module eigengenes with traits of interest, including sex, feather type (back vs. chest), chest feather color (cream vs. black), back feather color (brown vs. red), unornamented versus T-implanted males (e.g., experimental testosterone treatment), and unornamented versus ornamented male (e.g., natural difference in testosterone concentrations).

We analyzed functional enrichment of biological process GO terms in WebGestalt (Liao et al. 2019) for each module identified by WGCNA, using *H. sapiens* as the reference. We used “over-representation analysis (ORA)” as our method of interest, “gene ontology: biological processes” as our functional database, and “genome protein coding” as our reference set. For each module, we identified genes in each module with the highest network connectivity assessed by module membership and only included genes in GO enrichment analysis that had a module membership ≥0.6 (supplementary table S9, Supplementary Material online). We also ran this same analysis using *G. gallus* as the reference (supplementary table S10, Supplementary Material online).

### Feather Carotenoid Analysis

We assessed the carotenoid content of feathers from the red back feathers and black chest feathers of three ornamented male red-backed fairywrens, the cream chest feathers of three unornamented male red-backed fairywrens, and the black chest feathers of three male white-shouldered fairywrens. We trimmed 1–2.5 mg of pigmented barbs from the distal end of individual feathers for each sample and extracted carotenoids using methanol as described previously (Toomey, Smith, et al. 2022). Briefly, we ground each sample in a tube with 1 ml of methanol and 0.1 g of 1-mm zirconia beads on a beadbug (Benchmark Sci. Inc.) at 4 kHz for 9 min, centrifuged each tube, and extracted the supernatant, then repeated these steps three additional times to ensure full extraction of carotenoids. We dried the supernatant collected for each sample under a stream of nitrogen gas and then dissolved the extracted carotenoids in 125 µl of mobile phase (44:44:12 acetonitrile:methanol:dichloromethane, vol:vol:vol) for reverse-phase HPLC analysis.

We injected 100 µl of each sample into an Agilent 1200 series HPLC fitted with a YMC carotenoid column (5.0 µm, 4.6 mm × 250 mm) that was warmed 30°C. We eluted samples at a constant flow rate of 1.2 ml/min, beginning with a mobile phase consisting of 44:44:12 acetonitrile:methanol:dichloromethane (vol:vol:vol) then ramping up to 35:35:30 from 11 to 21 min, and holding at that condition through 35 min. Sample elution was monitored using a ultraviolet–visible (UV–Vis) photodiode array detector at 445 and 480 nm. Major carotenoids were identified by comparison to authentic standards (a gift of DSM Inc.) or published accounts (Toomey et al. 2022b). We quantified carotenoid content by comparison to external standard curves of astaxanthin for ketocarotenoids and zeaxanthin for other carotenoid types. Detection limits were 0.000203 µg for zeaxanthin and 0.0003 µg for the ketocarotenoid astaxanthin. Spectra peaks for one red back feather sample are shown in supplementary figure S2, Supplementary Material online and results of the HPLC analysis, including carotenoid identity, and the spectra peaks associated with those can be found in supplementary table S11, Supplementary Material online.

## Supplementary Material

msad056_Supplementary_Data

## Data Availability

The red-backed fairywren reference genome is available on NCBI under BioProject PRJNA934711. The transcriptomic raw data are available on NCBI under BioProject PRJNA900392.
