# Supplementary material for: Testosterone Coordinates Gene Expression Across Different Tissues to Produce Carotenoid-Based Red Ornamentation
Source: Mol Biol Evol. 2023 Mar 13;40(4):msad056. doi: 10.1093/molbev/msad056 (PMC10072822; doi:10.1093/molbev/msad056)
Supplement: msad056_Supplementary_Data [file msad056_supplementary_data.zip › supplementary_figures_final.pdf]

Supplementary figures for “Testosterone coordinates gene expression across different tissues to produce carotenoid-based red ornamentation.”

Sarah Khalil, Erik D. Enbody, Carolina Frankl-Vilches, Joseph F. Welklin, Rebecca E. Koch, Matthew B. Toomey, Simon Yung Wa Sin, Scott V. Edwards, Manfred Gahr, Hubert Schwabl, Michael S. Webster, Jordan Karubian

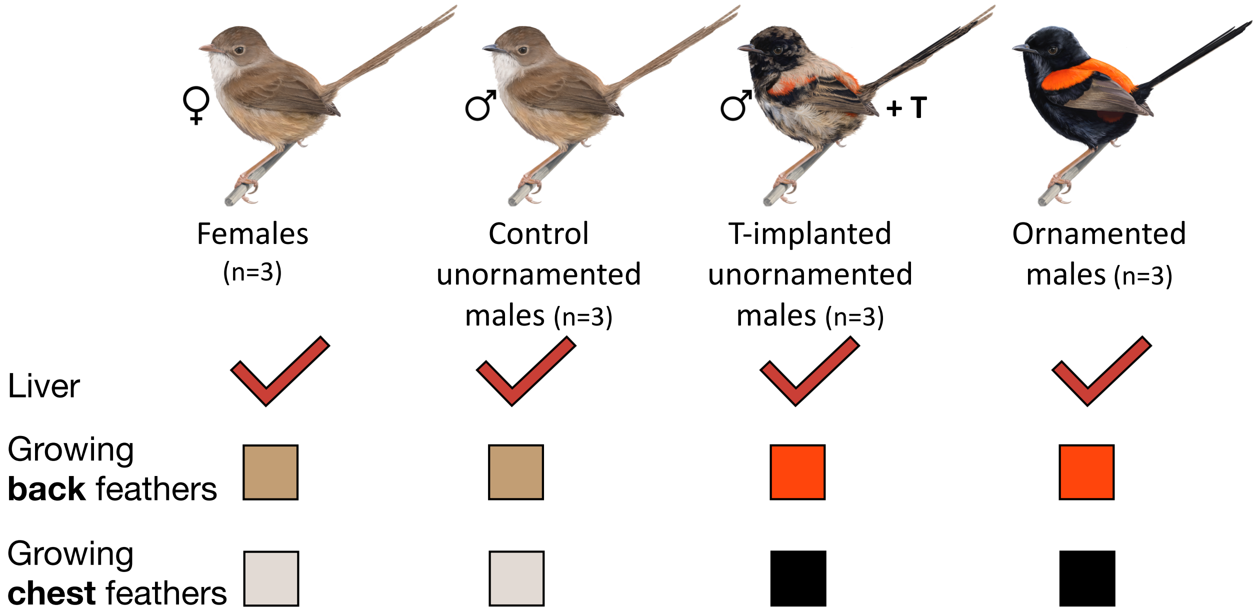

Figure S1– Sampling scheme of tissues sequenced. Colors of boxes represent the color of the feather for each respective phenotype.

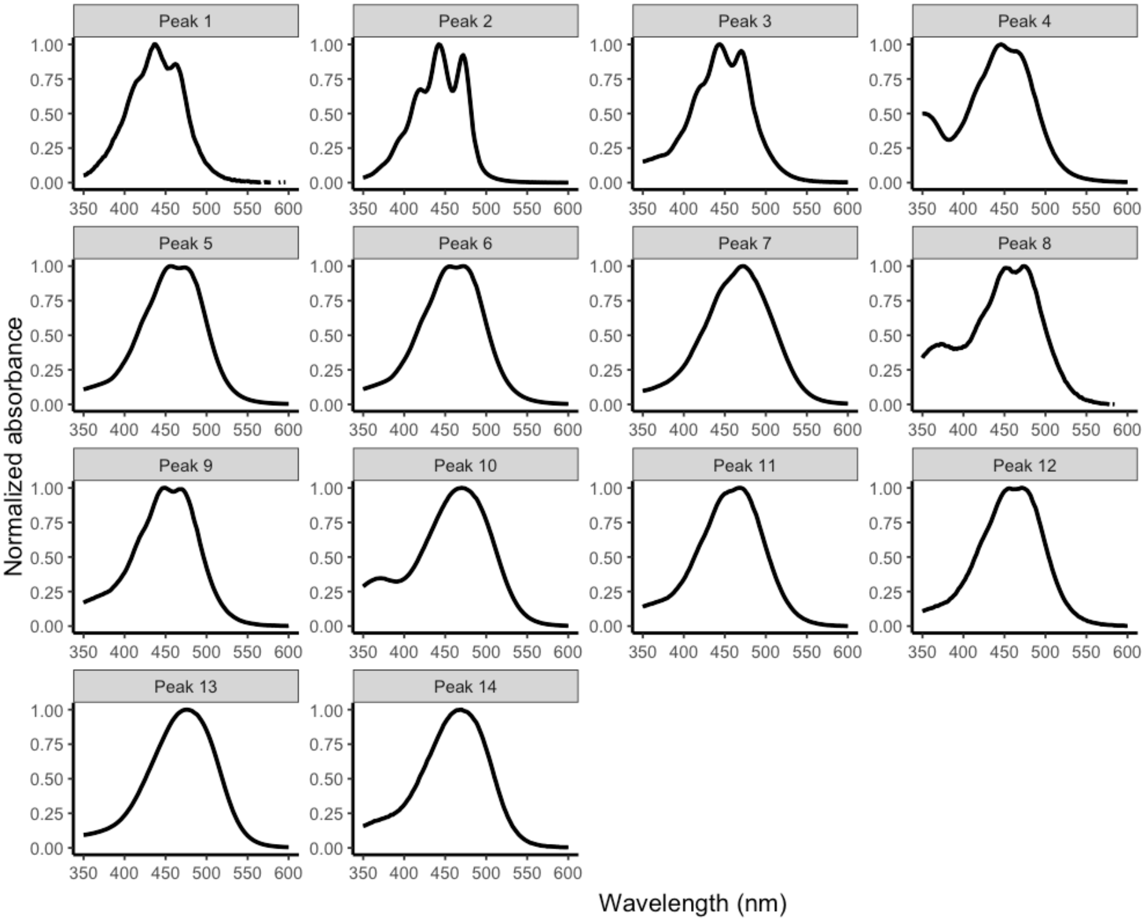

Figure S2 - Spectra associated with the main peaks we found in our HPLC analysis (i.e. any peaks that comprised >1% of the total carotenoids detected in the sample). Peaks are extracted from the top sample in Table S11 - the red back of male B10.

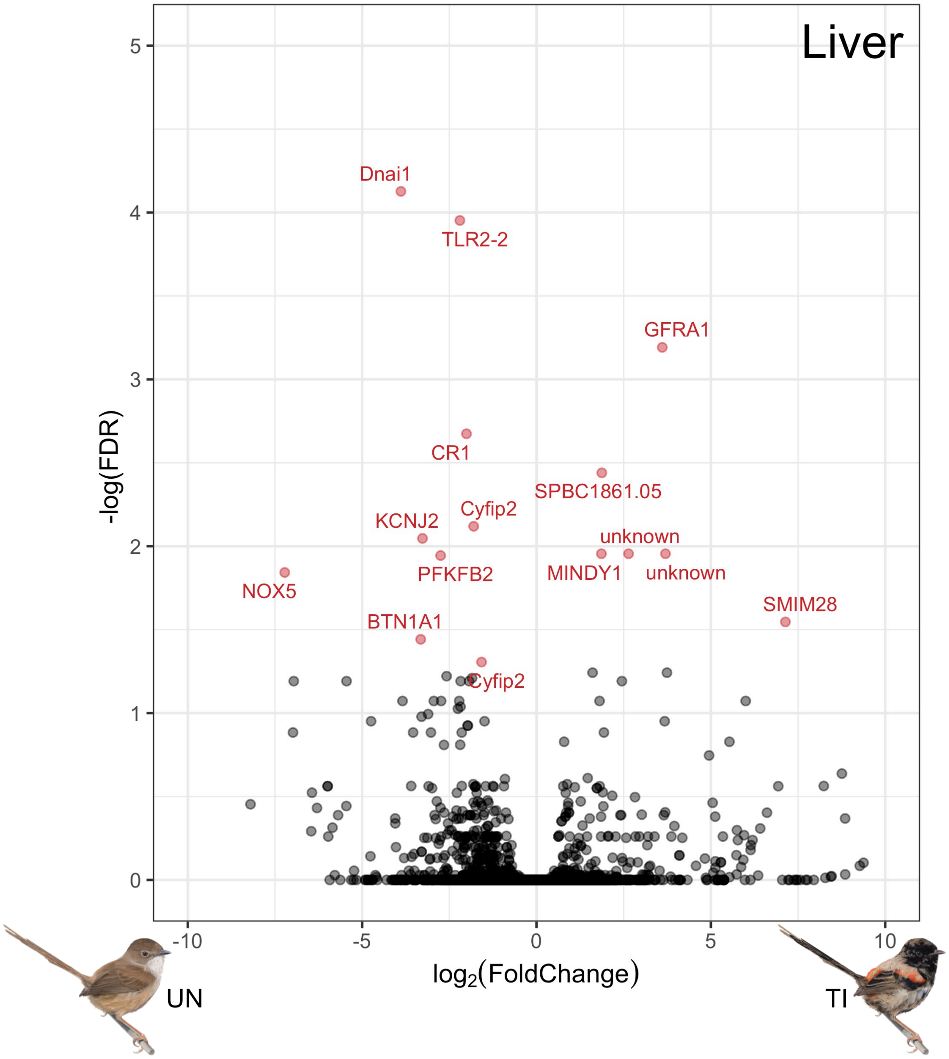

Figure S3 – Volcano plot of differential expression of genes in liver between control unornamented (UN) males and testosterone-implanted (TI) males, with significantly overexpressed genes (p.adj<0.05) plotted in red.

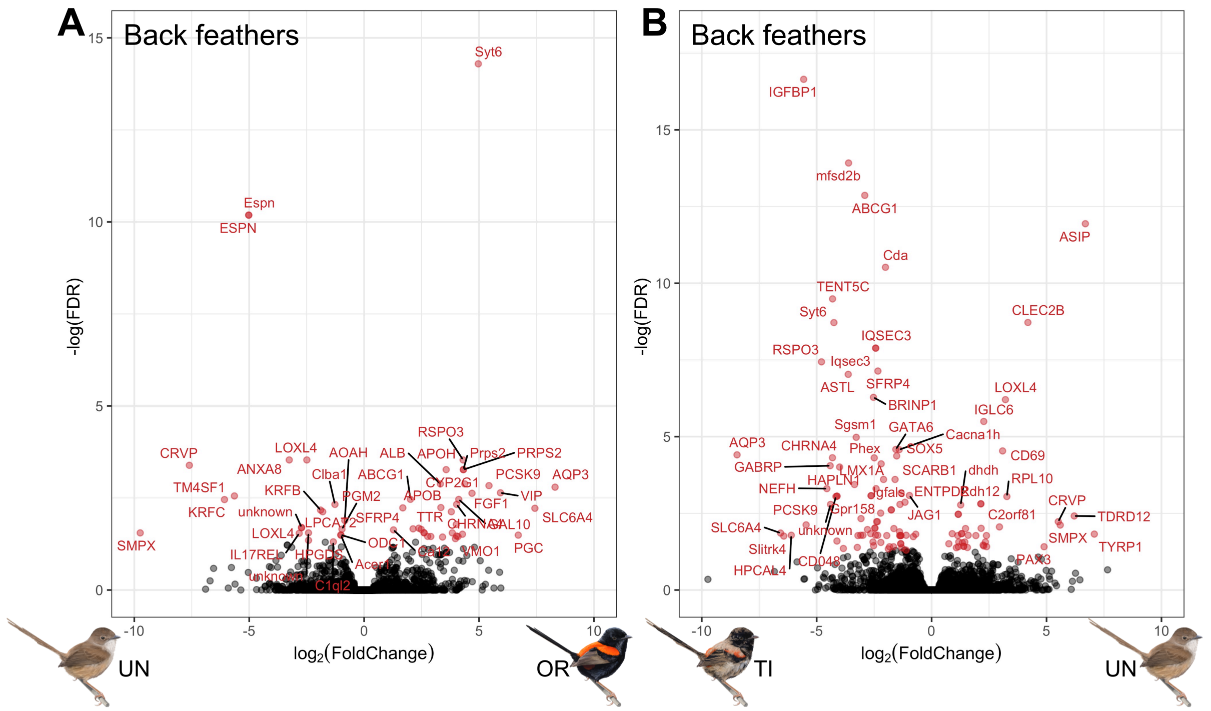

Figure S4 – Volcano plots of differential expression of genes in back feathers between **A.** unornamented (UN) males (brown feathers) and ornamented (OR) males (red feathers), and between **B.** unornamented (UN) males (brown feathers) and testosterone-implanted (TI) males (red feathers). Significantly overexpressed genes (p.adj<0.05) are plotted in red.

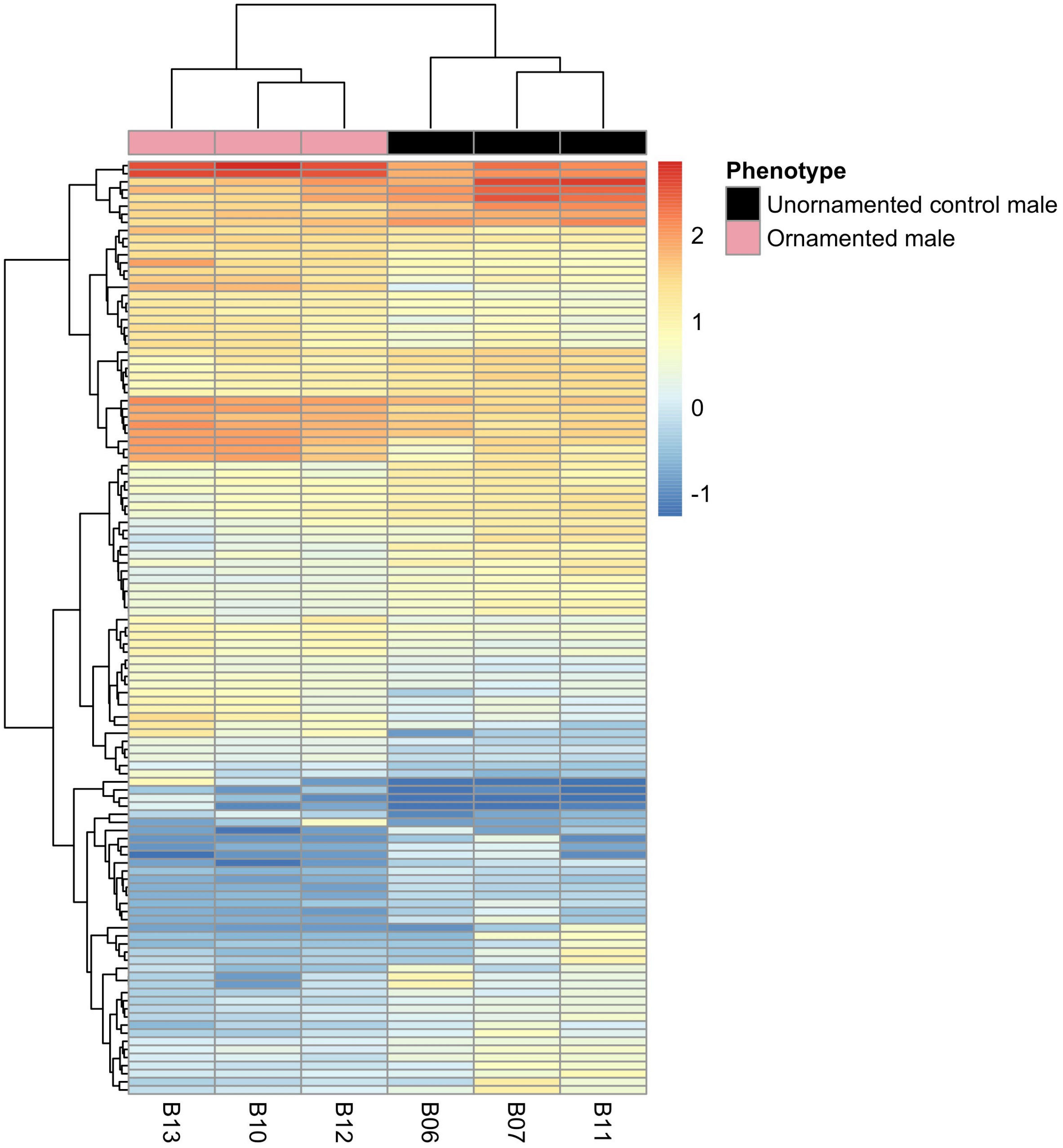

Figure S5 – Heatmap of all the genes significantly differentially expressed between unornamented control and ornamented males in **the liver**. Each row represents a gene, and each column represents an individual (sample number is at the bottom). Color represents Z-transformed gene counts for each gene in each individual. Gene names are not shown because they would not fit in the figure. Individuals and genes self-cluster by similarity (represented by the dendograms). Note that unornamented and ornamented males self-cluster within each other, suggesting their gene expression patterns are similar to each other. Heatmaps were created using the pheatmap package in R (Raivo 2012).

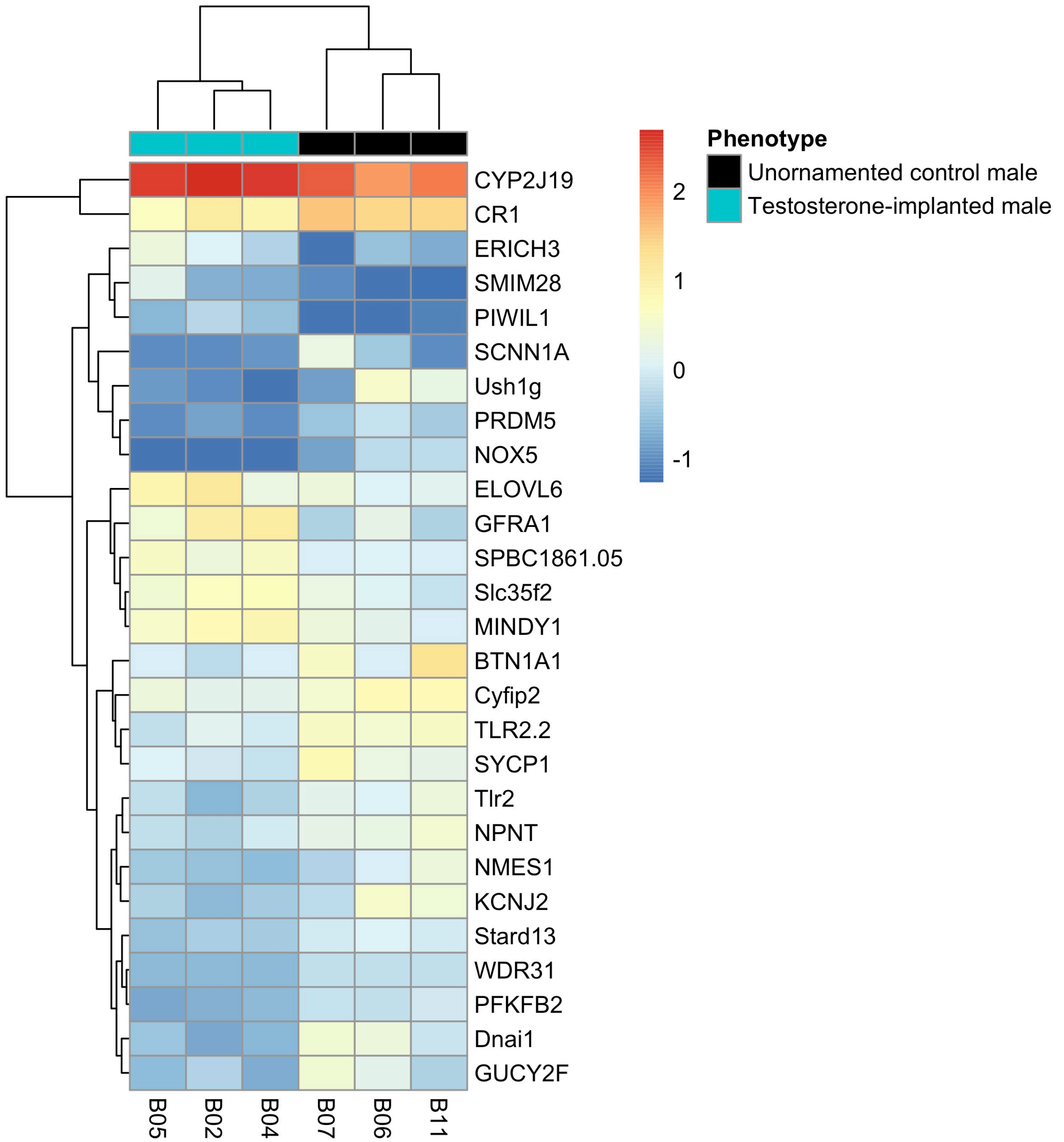

Figure S6 – Heatmap of all the genes significantly differentially expressed between unornamented control and testosterone-implanted males in **the liver**. Each row represents a gene, and each column represents an individual (sample number is at the bottom). Color represents Z-transformed gene counts for each gene in each individual. Individuals and genes self-cluster by similarity (represented by the dendograms). Note that unornamented and testosterone-implanted males self-cluster within each other, suggesting their gene expression patterns are similar to each other. Heatmaps were created using the pheatmap package in R (Raivo 2012).

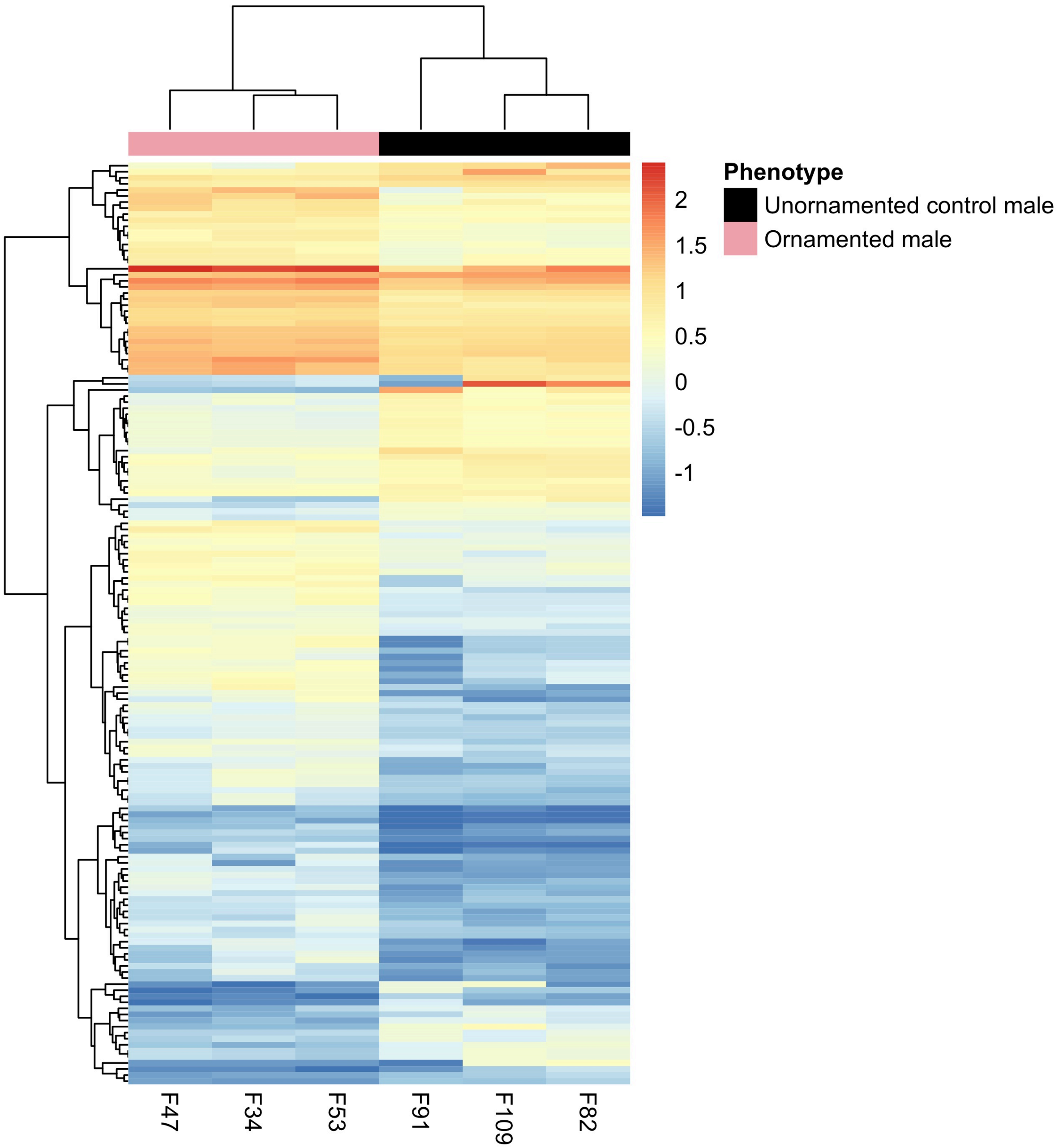

Figure S7 – Heatmap of all the genes significantly differentially expressed between unornamented control and ornamented males in **back feathers**. Each row represents a gene, and each column represents an individual (sample number is at the bottom). Color represents Z-transformed gene counts for each gene in each individual. Gene names are not shown because they would not fit in the figure. Individuals and genes self-cluster by similarity (represented by the dendograms). Note that unornamented and ornamented males self-cluster within each other, suggesting their gene expression patterns are similar to each other. Heatmaps were created using the pheatmap package in R (Raivo 2012).

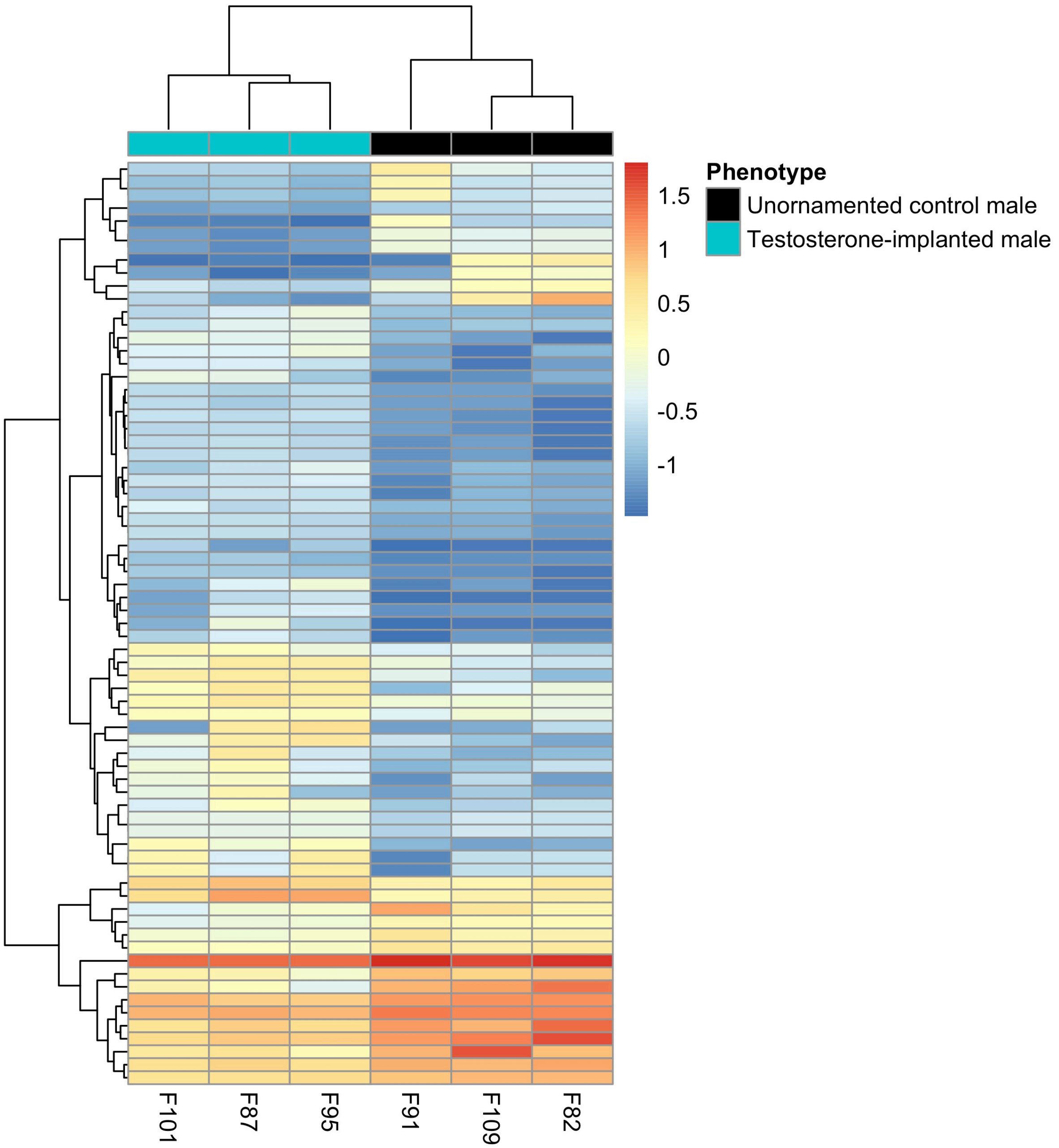

Figure S8 – Heatmap of all the genes significantly differentially expressed between unornamented control and testosterone-implanted males in **back feathers**. Each row represents a gene, and each column represents an individual (sample number is at the bottom). Color represents Z-transformed gene counts for each gene in each individual. Gene names are not shown because they would not fit in the figure. Individuals and genes self-cluster by similarity (represented by the dendograms). Note that unornamented and testosterone-implanted males self-cluster within each other, suggesting their gene expression patterns are similar to each other. Heatmaps were created using the pheatmap package in R (Raivo 2012).

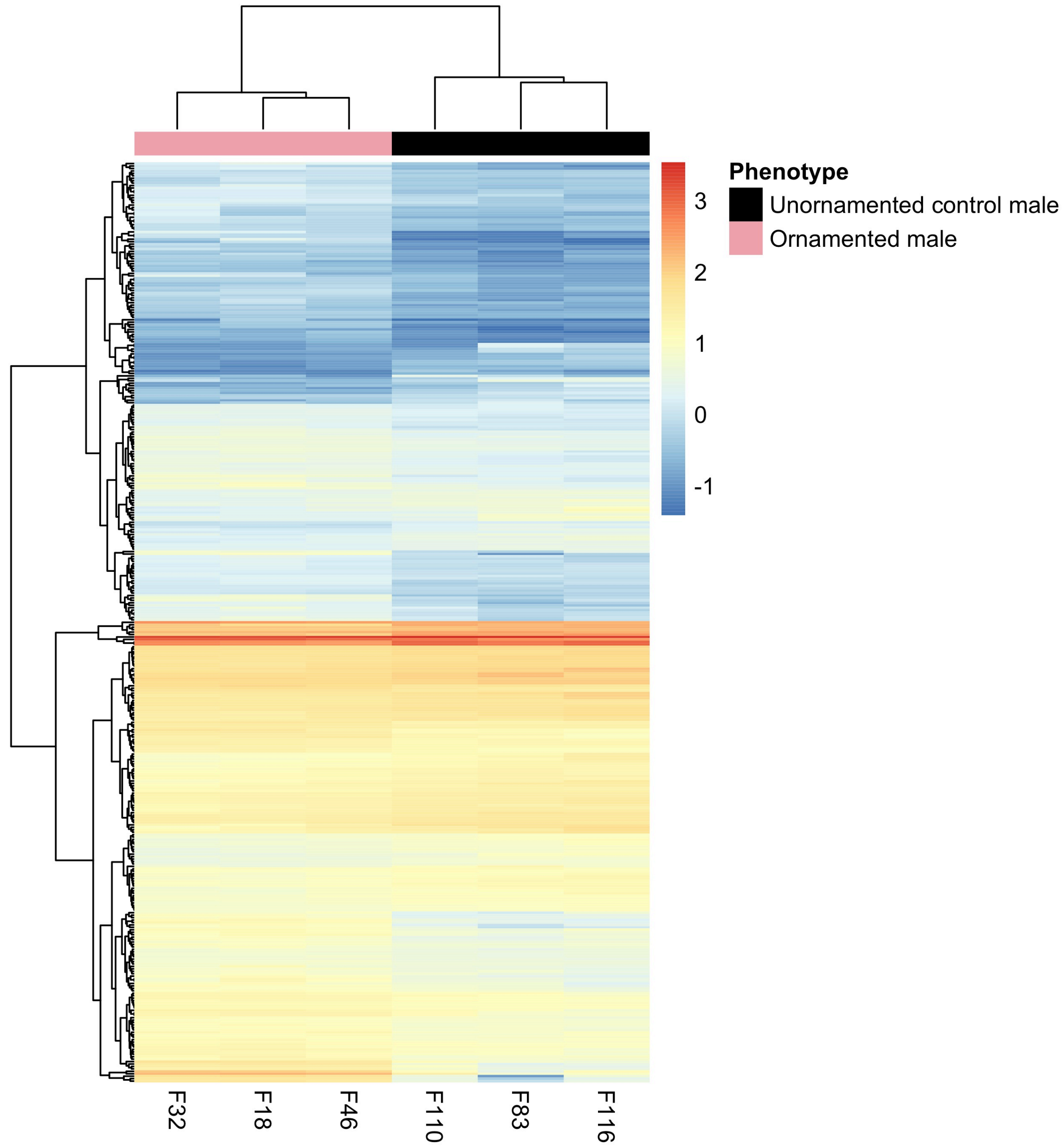

Figure S9 – Heatmap of all the genes significantly differentially expressed between unornamented control and ornamented males in **chest feathers**. Each row represents a gene, and each column represents an individual (sample number is at the bottom). Color represents Z-transformed gene counts for each gene in each individual. Gene names are not shown because they would not fit in the figure. Individuals and genes self-cluster by similarity (represented by the dendograms). Note that unornamented and ornamented males self-cluster within each other, suggesting their gene expression patterns are similar to each other. Heatmaps were created using the pheatmap package in R (Raivo 2012).

References:

Kolde, Raivo. "Pheatmap: pretty heatmaps." *R package version* 1.2 (2012): 726.
